# Association Between Gastrointestinal-Related Quality of Life and Frailty Using Baseline Data of the Prospective Cohort Study (JUSTICE-TOKYO Study)

**DOI:** 10.3390/diagnostics15010015

**Published:** 2024-12-25

**Authors:** Daisuke Asaoka, Osamu Nomura, Koji Sugano, Kei Matsuno, Hiroyuki Inoshita, Nobuto Shibata, Hideki Sugiyama, Noemi Endo, Yoshiyuki Iwase, Miyuki Tajima, Naoko Sakuma, Megumi Inoue, Mariko Nagata, Taeko Mizutani, Mizuki Ishii, Sachi Iida, Yoshiko Miura, Yuji Nishizaki, Naotake Yanagisawa, Tsutomu Takeda, Akihito Nagahara, Katsumi Miyauchi

**Affiliations:** 1 Department of Gastroenterology, Juntendo Tokyo Koto Geriatric Medical Center, Tokyo 136-0075, Japan; onomura@juntendo.ac.jp; 2 Department of Respiratory Medicine, Juntendo Tokyo Koto Geriatric Medical Center, Tokyo 136-0075, Japan; ksugano@juntendo.ac.jp (K.S.); kmatsuno@juntendo.ac.jp (K.M.); 3 Department of Nephrology, Juntendo Tokyo Koto Geriatric Medical Center, Tokyo 136-0075, Japan; ino-hi@juntendo.ac.jp; 4 Department of Psychiatry, Juntendo Tokyo Koto Geriatric Medical Center, Tokyo 136-0075, Japan; nshibata@juntendo.ac.jp (N.S.); h.sugiyama.ad@juntendo.ac.jp (H.S.); n.endo.hs@juntendo.ac.jp (N.E.); 5 Department of Orthopedic Surgery, Juntendo Tokyo Koto Geriatric Medical Center, Tokyo 136-0075, Japan; iwase@juntendo.ac.jp; 6 Department of Pharmacy, Juntendo Tokyo Koto Geriatric Medical Center, Tokyo 136-0075, Japan; mitajima@juntendo.ac.jp (M.T.); n.sakuma.rg@juntendo.ac.jp (N.S.); m.inoue.gm@juntendo.ac.jp (M.I.); nagata_mariko@juntendo.gmc.ac.jp (M.N.); 7 Department of Nursing, Juntendo Tokyo Koto Geriatric Medical Center, Tokyo 136-0075, Japan; t-mizutani@juntendo.ac.jp (T.M.); mz-ishii@juntendo.ac.jp (M.I.); sahirai@juntendo.ac.jp (S.I.); 8 Department of Nutrition, Juntendo Tokyo Koto Geriatric Medical Center, Tokyo 136-0075, Japan; yoshi-m@juntendo.ac.jp; 9 Medical Technology Innovation Center, Juntendo University, Tokyo 113-8421, Japan; ynishiza@juntendo.ac.jp (Y.N.); n-yanagisawa@juntendo.ac.jp (N.Y.); 10 Department of Gastroenterology, Juntendo University School of Medicine, Tokyo 113-8421, Japan; t-takeda@juntendo.ac.jp (T.T.); nagahara@juntendo.ac.jp (A.N.); 11 Department of Cardiology, Juntendo Tokyo Koto Geriatric Medical Center, Tokyo 136-0075, Japan; ktmmy@juntendo.ac.jp

**Keywords:** frailty, sarcopenia, Mini Mental State Examination, Geriatric Depression Scale, EuroQol-Visual Analogue Scale, Izumo scale

## Abstract

**Objective:** To determine the prevalence of frailty and examine its association with gastrointestinal-related quality of life (QOL) among older outpatients in a geriatric hospital. **Methods:** This cross-sectional study involved 1042 outpatients (age: ≥65 years) diagnosed using the revised Japanese version of the cardiovascular health study criteria. Data collection was performed by a multidisciplinary team. **Results:** Of the 1039 eligible subjects (male: *n* = 460 [44.3%]; mean age: 78.2 ± 6.1 years), 172 (16.6%) had frailty (male: *n* = 77 [44.8%]; mean age: 80.9 ± 6.2 years). The multivariate analysis revealed that age (odds ratio [OR]: 1.070, *p* < 0.001), QOL (OR: 0.982, *p* = 0.009), a history of falls (OR: 1.702, *p* = 0.029), sarcopenia (OR: 4.708, *p* < 0.001), steroid use (OR: 3.741, *p* = 0.001), analgesic drug use (OR: 2.056, *p* = 0.014), Mini Mental State Examination (OR: 0.915, *p* = 0.011), Geriatric Depression Scale 15 (OR: 1.101, *p* = 0.008), fullness-related QOL score (OR: 1.119, *p* = 0.010), chronic obstructive pulmonary disease assessment test (OR: 1.048, *p* = 0.007), and 10-item Eating Assessment Tool (OR: 1.071, *p* = 0.009) were related to frailty. **Conclusions:** The prevalence rate of frailty in older outpatients at a university hospital specializing in geriatric medicine was higher than that previously reported in community-dwelling individuals. Our study clarified that the fullness-related QOL score was related to frailty.

## 1. Introduction

As Japan enters the era of 100-year life expectancy, the management of bedridden elderly people and those requiring nursing care is becoming a challenge. Interventions for frailty are gaining attention as a means of preventing bedridden patients and those requiring long-term care. The term frailty refers to a vulnerable state that increases the high risk of adverse health outcomes among older adults [1,2]. Recently, the revised Japanese version of the Cardiovascular Health Study criteria was published [3]. However, numerous publications have reported that the prevalence of frailty varies widely across study designs, populations, and settings. Although there are many causes of undernutrition in the elderly, the process of oral intake and digestion of nutrients is important. Functional diseases that cause reduced quality of life (QOL) due to gastrointestinal symptoms include non-erosive reflux disease, functional dyspepsia, and irritable bowel syndrome.

An abdominal QOL scale (Izumo scale) has been developed to assess overall gastrointestinal symptoms [4]. In Japan, few studies have investigated the association between gastrointestinal-related QOL and sarcopenia among older outpatients in a geriatric hospital. In addition, our university hospitals specializing in the elderly have sufficient staff for a comprehensive diagnosis of frailty, comprising doctors and nurses as well as pharmacists, psychologists, dieticians, radiologists, dentists, and other professionals. The purpose of this study was to determine the prevalence rate of frailty and examine its association with gastrointestinal-related QOL among older outpatients in a geriatric hospital through a multidisciplinary approach.

## 2. Materials and Methods

### 2.1. Study Design

We conducted a single-center, cross-sectional investigation using baseline data from the JUntendo Sarcopenia regisTratIon of exploring for prediCtors and prognosis in Elderly in TOKYO (JUSTICE-TOKYO) study [5]. The ongoing JUSTICE-TOKYO study involves older outpatients (age: ≥65 years) who presented at Juntendo Tokyo Koto Geriatric Medical Center between November 2020 and November 2021. This study enrolled only Japanese participants. The investigation involves a 4-year follow-up, and analyses (i.e., survival, incidence of falls, hospitalization, and skeletal muscle mass measurements) are conducted annually; this study will be completed in 2025. At the time of enrollment, baseline data (i.e., patient profile, comorbidities, questionnaires, physical and skeletal muscle mass measurements, physiological function test results, and nutritional status) were collected in a multidisciplinary setting. Data were prospectively entered into the Research Electronic Data Capture (REDCap) system [6].

### 2.2. Exclusion Criteria

The exclusion criteria were inability to walk independently because of severe osteoarthritis or neuromuscular disease, immobility, presence of delirium tremens, and history of acute (within 6 months) cerebrovascular, gastrointestinal, renal, coronary, hepatic, and respiratory events. Patients who could not be interviewed by questionnaire and those with expected life expectancies < 1 year because of malignant disease were also excluded.

### 2.3. Measurement of Baseline Variables

Patients for whom the information below was available at registration were included in the analysis:

(1) Patient profile, i.e., age, sex, body mass index, Brinkman Index, drinking habits (defined as follows: 0 = rarely, 1 = 1–4 days/week, 2 = 5–7 days/week), phase angle (PhA), QOL (EuroQol-Visual Analogue Scale [EQ-VAS]), history of falls, history of daycare use, and social frailty [7,8,9]. Body composition was determined based on resistance to a multi-frequency alternating current applied to the body and analysis of impedance characteristics (e.g., capacitive reactance and PhA) using an MC-780A analyzer (TANITA, Tokyo, Japan). Patients with pacemaker implantation did not undergo BIA evaluation. QOL was evaluated with the EQ-VAS life quality questionnaire (scores of 0 and 100 indicate worst and perfect health, respectively). Moreover, nurses and nutritionists evaluated anthropocentric measures, physiological performance, walking speed, etc.

(2) Comorbidities (i.e., history of cerebral infarction/hemorrhage, myocardial infarction, hospitalization for heart failure, interstitial pneumonia, malignant disease, hypertension, diabetes mellitus [DM], atrial fibrillation, and osteoporosis). The percentage of young adult mean T-scores were measured through dual X-ray absorptiometry of the lumbar spine (L2–L4) and total hip. Dual X-ray absorptiometry scanning was performed using a Prodigy Advance scanner (GE Healthcare, Madison, WI, USA). Osteoporosis was diagnosed according to the criteria of the Japanese Society for Bone and Mineral Research [10]. Sarcopenia was defined based on the diagnostic algorithm recommended by the Asian Working Group for Sarcopenia: 2019 Consensus Update on Sarcopenia Diagnosis and Treatment [11]. Frailty was diagnosed based on the criteria of the Japanese version of the Cardiovascular Health Study (adapted from the original Cardiovascular Health Study criteria) [3]. The criteria include unintentional weight loss, fatigue, inactivity, low grip strength, and slow gait speed. Unintentional weight loss was defined as a decrease in body weight of more than 2 kg in the past 6 months without any particular cause. Fatigue was defined as self-reported exhaustion and was assessed using the following question: “In the past 2 weeks, have you felt tired without a reason?” Activity level was evaluated using the following questions: “Do you engage in moderate levels of physical exercise or sports in an effort to maintain health” and “Do you engage in low levels of physical exercise in an effort to maintain health”. If a subject answered “No” to both of these questions, then we considered their physical activity to be low. Low grip strength was defined as a grip strength of <28 kg in men and <18 kg in women. A slow gait speed was defined as a gait speed of <1.0 m/s. In this study, subjects with impairments in three or more of the five criteria were categorized into the frailty group, while those with fewer than three criteria were categorized into a non-frailty group.

(3) Pharmacological therapy (i.e., statins, acid secretion suppressants, laxatives, steroids, analgesic drugs, antidementia drugs, and antipsychotic drugs) and administration of oral medication. Pharmacists extracted these data from the personal health notebook of patients.

(4) Neuropsychological examination performed by psychiatrists (i.e., Mini Mental State Examination [MMSE] and 15-item Geriatric Depression Scale [GDS-15) [12,13].

(5) Gastrointestinal-related QOL (Izumo scale) scores (reflux, upper abdominal pain, fullness, constipation, and diarrhea-related QOL score) [4]. The Izumo scale is a 15-item self-administered questionnaire assessing five domains, namely reflux, upper abdominal pain, fullness, constipation, and diarrhea. A Likert scale (0–5) is used to obtain a score for each item. Domain-specific QOL impairment is scored from 0 (no impairment) to 15 (severe impairment).

(6) Severity of constipation using the Constipation Scoring System (CSS) score [14] comprised of eight items (i.e., frequency of bowel movements, painful evacuation, incomplete evacuation, abdominal pain, length of time per attempt, assistance for evacuation, unsuccessful attempts at evacuation per 24 h, and duration of constipation). The overall CSS score ranges from 0–30, with higher scores indicating worse constipation symptoms. The CSS and Izumo scales were evaluated by gastroenterologists.

(7) Pulmonary function (i.e., arterial oxygen saturation, chronic obstructive pulmonary disease assessment test [CAT] results, restricted ventilatory impairment, and obstructive ventilatory impairment) [15,16]. The impact of chronic obstructive pulmonary disease (COPD) on health status was evaluated using the CAT, which consists of eight items. A CAT score of ≥10 indicates a high symptomatic level. Pulmonary function tests were carried out with the Minato System 21 (Minato Medical Science Co., Ltd., Osaka, Japan) to measure vital capacity, forced vital capacity, and forced expiratory volume in 1 s. Respiratory physicians evaluated the CAT, 10-item Eating Assessment Tool (EAT10), and pulmonary function tests.

(8) Nutritional status (hypozincemia, CONtrolling NUTritional status [CONUT] score, and Dietary Variety Score [DVS]). In this study, we defined hypozincemia as treatment with zinc acetate hydrate (Novelzin^®^ Tablets, Nobelpharma K.K., Tokyo, Japan) or serum zinc levels < 80 µg/dL. Zinc is an essential trace element in living organisms. Hypozincemia is related to appetite loss [17], depression [18], and taste abnormality [19], and these symptoms have been linked to hypoalimentation. The objective nutritional status was determined using the CONUT score (ranging from 0–12). The CONUT score was calculated based on serum albumin levels, total cholesterol levels, and total lymphocyte count [20]. A nutritional assessment based solely on a subjective comprehensive assessment may miss the risk of low nutritional risk associated with inflammation without weight loss. The CONUT score, a nutritional index derived from blood laboratory values, is a simple and useful nutritional indicator. The DVS comprises 10 food-based components [21].

(9) Oral function (Oral Frailty Index-8 [OFI-8] [22] and EAT10 [23]). Oral frailty was defined by an OFI-8 score ≥ 4 points. Dysphagia was assessed by the EAT10.

Data were obtained within 3 months following registration. It took approximately 2 h to fully evaluate all parameters per participant in the manuscript.

### 2.4. Statistical Analysis

We formed two groups (frailty and non-frailty) and performed univariate and multivariate analyses to compare risk factors for sarcopenia. Univariate analyses were carried out using a χ^2^ test or Student’s *t*-test for categorical and continuous variables, respectively. Independent variables with *p*-values < 0.20 in the univariate analysis were included in the multivariate logistic regression analysis. Odds ratios (ORs) and 95% confidence intervals were also used to identify associations. Statistical analyses were performed using SPSS version 28 software (IBM Corporation, Armonk, NY, USA). *p*-values < 0.05 indicate statistical significance.

## 3. Results

### 3.1. Patient Characteristics

Table 1 shows the characteristics of the 1039 subjects included in this study (male: *n* = 460 [44.3%]; mean age: 78.2 ± 6.1 years; mean body mass index: 22.9 ± 3.7 kg/m^2^). Of those, 172 and 867 were diagnosed with frailty and non-frailty, respectively.

### 3.2. Frailty and Covariates in the Univariate Analysis

Table 2 shows the prevalence of frailty and the association between sarcopenia and covariates according to the univariate analysis. Significant differences between the frailty and non-frailty groups were observed in age, PhA, QOL, history of falls, history of daycare use, social frailty, cerebral infarction/hemorrhage, myocardial infarction, interstitial pneumonia, diabetes mellitus, osteoporosis, sarcopenia, statin, laxative, steroid, and analgesic drugs, antipsychotic drugs, number of oral medicines, MMSE, GDS-15, reflux, upper abdominal pain, fullness, constipation, diarrhea-related QOL score, CSS score, CAT, restricted ventilatory impairment, hypozincemia, CONUT score, DVS, oral frailty, and EAT10.

### 3.3. Frailty and Covariates in the Multivariate Analysis

In the multivariate analysis, age (OR: 1.070, *p* < 0.001), QOL (OR: 0.982, *p* = 0.009), history of falls (OR: 1.702, *p* = 0.029), sarcopenia (OR: 4.708, *p* < 0.001), steroids (OR: 3.741, *p* = 0.001), analgesic drugs (OR: 2.056, *p* = 0.014), MMSE (OR: 0.915, *p* = 0.011), GDS-15 (OR: 1.101, *p* = 0.008), fullness-related QOL score (OR: 1.119, *p* = 0.010), CAT (OR: 1.048, *p* = 0.007), and EAT10 (OR: 1.071 *p* = 0.009) were related to frailty (Table 3).

## 4. Discussion

This was the first large cross-sectional study aimed at investigating, in a multidisciplinary setting, the prevalence and risk factors of frailty in older outpatients of a university hospital specializing in geriatric medicine. The results demonstrated that age, QOL, history of falls, sarcopenia, steroids, analgesic drugs, MMSE, GDS-15, fullness-related QOL score, CAT, and EAT10 were related to frailty.

According to a systematic review and meta-analysis, the prevalence of frailty among Japanese community-dwelling older individuals was 7.4% [24]. The prevalence of frailty in this study was higher (16.6%) than that recorded among community-dwelling individuals in Japan. This difference might be due to the higher number of patients with multiple severe diseases treated at the geriatric hospital. In addition, the mean age of subjects in our study was higher (78.2 ± 6.1 years) than that reported in other studies [24]. Another systematic review and meta-analysis of community-dwelling older individuals revealed that Asian populations are exposed to more risk factors for frailty compared with other populations [25]. However, based on a systematic review and meta-analysis of geriatric hospital inpatients, the prevalence rate of frailty and pre-frailty was 47.4% and 25.8%, respectively [26]. In the present study, the prevalence rate of frailty was between those recorded in the geriatric hospital and the general local population, as the patients were treated in a university hospital specializing in geriatric medicine.

As shown in Table 2, sex was not associated with significant differences in the prevalence of frailty. Following stratification by both age and sex, a systematic review and meta-analysis in Japan revealed that the prevalence rate of frailty increased with age in both sexes [24]. In a systematic review and meta-analysis, the pooled prevalence rate of frailty among female and male geriatric hospital inpatients was 51.9% and 47%, respectively, and differences in the prevalence of frailty between sexes were not statistically significant [26].

In the present study, frailty was significantly associated with several factors, including age, QOL, history of falls, sarcopenia, MMSE, GDS-15, CAT, and EAT10. A systematic review and meta-analysis identified older age, depression, and a history of falls as risk factors for frailty in older individuals in Asia [25]. Namioka et al. suggested that physical frailty is frequent in older and female Alzheimer’s disease patients with comorbidities [27]. A systematic review and meta-analysis revealed a clear association of frailty with lower QOL across a range of constructs [28]. By studying community-dwelling populations, Nishiguchi et al. reported that frail individuals were at a significantly higher risk of developing sarcopenia than non-frail individuals [29]. Kagiali et al. revealed higher CAT scores in patients with frailty versus those without frailty [30].

Regarding the use of therapeutic agents, steroid and analgesic drugs were related to frailty. Ryu et al. showed that cumulative long-term exposure to oral corticosteroid therapy was linked to a higher prevalence rate of frailty and muscle weakness in elderly patients with asthma [31]. Concerning gastrointestinal-related QOL scores, reflux, upper abdominal pain, fullness, constipation, and diarrhea-related QOL scores were lower in frailty groups than in non-frailty groups in the univariate analysis. However, this study clarified that the fullness-related QOL score was related to frailty in the multivariate analysis. In a previous systematic review and meta-analysis, the association between frailty and lower QOL across a range of constructs is clear [28]. In this study, frailty was also significantly associated with lower QOL (EQ-5D). Undernutrition is an important cause of frailty. The food we eat is digested in the gastrointestinal tract and then absorbed in the intestine to become nutrients. Therefore, we focused on the association between gastrointestinal quality of life (QOL) and frailty because poor QOL in the gastrointestinal tract can cause undernutrition. In outpatients aged ≥65 years and treated at the gastroenterology department of a hospital, high Frequency Scale for the Symptoms of Gastroesophageal Reflux Disease scores and high CSS scores were associated with frailty [32]. Among the various gastrointestinal symptoms, fullness-related symptoms may particularly have reduced appetite and decreased dietary variety, leading to undernutrition.

The present analysis has several limitations. Firstly, this study included only outpatients aged ≥65 years treated at the department of internal medicine of a single university hospital specializing in geriatric medicine. Secondly, we did not investigate other background variables (e.g., exercise routines, dietary pattern, occupations or careers, education level, and marital status). Consequently, the present data may not be generalizable to older individuals, and the results may be overestimated due to unhealthy subject bias.

## 5. Conclusions

In conclusion, this study showed a higher prevalence rate of frailty in outpatients in a geriatric hospital than that previously recorded in community-dwelling elderly individuals. The present findings clarified that among the various gastrointestinal symptoms, the fullness-related QOL score was particularly related to frailty. Future intervention studies assessing the effect of frailty improvement on abdominal symptoms are warranted.

## Figures and Tables

**Table 1:** Clinical characteristics of the study subjects (*n* = 1039).

Subject Profile	
**Age (years)**	78.2 ± 6.1
**Sex**	
**Male**	460 (44.3)
**Female**	579 (55.7)
**Body mass index (kg/m^2^)**	22.9 ± 3.7
**Brinkman Index**	358.8 ± 613.1
**Alcohol**	0.5 ± 0.8
**Phase angle (degree)**	−4.7 ± 0.8
**QOL (EQ-5D score)**	75.0 ± 17.0
**SpO^2^**	97.2 ± 2.4
**CAT score**	8.6 ± 6.6
**Gastrointestinal-related QOL**	
**Reflux**	1.8 ± 2.4
**Upper abdominal pain**	1.1 ± 2.0
**Fullness**	1.6 ± 2.4
**Constipation**	2.2 ± 2.6
**Diarrhea**	2.1 ± 2.6
**Severity of constipation**	
**Constipation Scoring System score**	3.5 ± 3.7
**Neuropsychological examination**	
**MMSE score**	26.5 ± 3.1
**GDS-15 score**	4.2 ± 3.0
**Health condition**	
**History of falls**	
**Yes**	205 (19.7)
**History of day care use**	
**Yes**	93 (9.0)
**Social frailty**	
**Yes**	681 (65.5)
**Comorbidities**	
**Cerebral infarction/hemorrhage**	
**Yes**	79 (7.6)
**Myocardial infarction**	
**Yes**	47 (4.5)
**Hospitalization for heart failure**	
**Yes**	42 (4.0)
**Interstitial pneumonia**	
**Yes**	55 (5.3)
**Malignancy disease**	
**Yes**	231 (22.2)
**Hypertension**	
**Yes**	607 (58.4)
**Diabetes mellitus**	
**Yes**	179 (17.2)
**Atrial fibrillation**	
**Yes**	87 (8.4)
**Osteoporosis**	
**Yes**	339 (32.6)
**Sarcopenia**	
**Yes**	222 (21.4)
**Use of therapeutic agents**	
**Statin**	
**User**	433 (41.7)
**Acid secretion suppressant**	
**User**	570 (54.9)
**Laxative**	
**User**	228 (21.9)
**Steroid**	
**User**	49 (4.7)
**Analgesic drugs**	
**User**	115 (11.1)
**Antidementia drugs**	
**User**	31 (3.0)
**Antipsychotic drug**	
**User**	259 (24.9)
**Number of oral medicines**	6.2 ± 3.5
**Pulmonary function**	
**Restricted ventilatory impairment**	
**Yes**	172 (16.6)
**Obstructive ventilatory impairment**	
**Yes**	261 (25.1)
**Nutritional status**	
**Hypozincemia**	
**Yes**	815 (78.4)
**CONUT score**	1.0 ± 1.2
**Dietary Variety Score**	3.7 ± 2.2
**Oral function**	
**Oral frailty**	
**Yes**	524 (50.4)
**EAT10 score**	1.6 ± 3.7

Data are presented as the number (%) or mean ± standard deviation. CAT—chronic obstructive pulmonary disease assessment test; CONUT—CONtrolling NUTritional status; EAT10—10-item Eating Assessment Tool; EQ-5D—EuroQol-five dimension; GDS-15—15-item Geriatric Depression Scale; MMSE—Mini Mental State Examination; QOL—quality of life; SpO^2^—arterial oxygen saturation.

**Table 2:** Association between frailty and covariates in the univariate analysis.

	Frailty Group	Non-Frailty Group	*p*-Value
**Covariates**	*n* = 172	*n* = 867	
	16.60%	83.40%	
**Patient characteristics**			
**Age (years)**	80.9 ± 6.2	77.7 ± 5.9	<0.001
**Sex**			0.886
**Male**	77 (44.8)	383 (44.2)	
**Female**	95 (55.2)	484 (55.8)	
**Body mass index (kg/m^2^)**	22.7 ± 3.9	23.0 ± 3.7	0.302
**Brinkman Index**	389.3 ± 619.0	352.8 ± 612.0	
**Alcohol**	0.4 ± 0.8	0.5 ± 0.8	0.135
**Phase angle (degree)**	−4.4 ± 1.1	−4.8 ± 0.8	<0.001
**QOL (EQ-5D score)**	64.9 ± 18.0	77.0 ± 16.0	<0.001
**SpO^2^**	97.1 ± 1.6	97.2 ± 2.5	0.536
**CAT score**	12.4 ± 7.7	7.8 ± 6.0	<0.001
**Gastrointestinal-related QOL**			
**Reflux**	2.4 ± 2.7	1.7 ± 2.3	<0.001
**Upper abdominal pain**	1.6 ± 2.5	1.0 ± 1.9	<0.001
**Fullness**	2.7 ± 3.0	1.4 ± 2.2	<0.001
**Constipation**	3.3 ± 3.0	2.0 ± 2.4	<0.001
**Diarrhea**	2.8 ± 3.1	1.9 ± 2.5	<0.001
**Severity of constipation**			
**Constipation Scoring System score**	5.1 ± 4.7	3.2 ± 3.4	<0.001
**Neuropsychological examination**			
**MMSE score**	25.4 ± 3.6	26.7 ± 2.9	<0.001
**GDS-15 score**	6.0 ± 3.2	3.8 ± 2.9	<0.001
**Health condition**			
**History of falls**			<0.001
**Yes**	57 (33.1)	148 (17.1)	
**History of day care use**			<0.001
**Yes**	32 (18.6)	61 (7.0)	
**Social frailty**			<0.001
**Yes**	140 (81.4)	541 (62.4)	
**Comorbidities**			
**Cerebral infarction/hemorrhage**			0.013
**Yes**	21 (12.2)	58 (6.7)	
**Myocardial infarction**			0.036
**Yes**	13 (7.6)	34 (3.9)	
**Hospitalization for heart failure**			0.197
**Yes**	10 (5.8)	32 (3.7)	
**Interstitial pneumonia**			0.01
**Yes**	16 (9.3)	39 (4.5)	
**Malignancy disease**			0.879
**Yes**	39 (22.7)	192 (22.1)	
**Hypertension**			0.051
**Yes**	112 (65.1)	495 (57.1)	
**Diabetes mellitus**			0.038
**Yes**	39 (22.7)	140 (16.1)	
**Atrial fibrillation**			0.857
**Yes**	15 (8.7)	72 (8.3)	
**Osteoporosis**			0.005
**Yes**	72 (41.9)	267 (30.8)	
**Sarcopenia**			<0.001
**Yes**	86 (50.0)	136 (15.7)	
**Use of therapeutic agents**			
**Statin**			0.048
**User**	60 (34.9)	373 (43.0)	
**Acid secretion suppressant**			0.051
**User**	106 (61.6)	464 (53.5)	
**Laxative**			0.001
**User**	54 (31.4)	174 (20.1)	
**Steroid**			<0.001
**User**	19 (11.0)	30 (3.5)	
**Analgesic drugs**			0.004
**User**	30 (17.4)	85 (9.8)	
**Antidementia drugs**			0.67
**User**	6 (3.5)	25 (2.9)	
**Antipsychotic drug**			<0.001
**User**	61 (35.5)	198 (22.8)	
**Number of oral medicines**	7.6 ± 5.9	5.9 ± 3.4	<0.001
**Pulmonary function**			
**Restricted ventilatory impairment**			<0.001
**Yes**	47 (27.3)	125 (14.4)	
**Obstructive ventilatory impairment**			0.968
**Yes**	43 (25.0)	218 (25.1)	
**Nutritional status**			
**Hypozincemia**			0.041
**Yes**	145 (84.3)	670 (77.3)	
**CONUT score**	1.4 ± 1.5	0.9 ± 1.1	<0.001
**Dietary Variety Score**	3.4 ± 2.3	3.7 ± 2.2	0.051
**Oral function**			
**Oral frailty**			<0.001
**Yes**	115 (66.9)	409 (47.2)	
**EAT10 score**	4.0 ± 6.3	1.1 ± 2.6	<0.001

Data are presented as the number (%) or the mean ± standard deviation. CAT—chronic obstructive pulmonary disease assessment test; CONUT—CONtrolling NUTritional status; EAT10—10-item Eating Assessment Tool; EQ-5D—EuroQol-five dimension; GDS-15—15-item Geriatric Depression Scale; MMSE—Mini Mental State Examination; QOL—quality of life; SpO^2^—arterial oxygen saturation.

**Table 3:** Association between frailty and covariates in the multivariate analysis.

Covariates	B	OR	95% CI	*p*-Value
**Subject profile**				
**Age (years)**	0.068	1.070	1.031–1.110	<0.001
**Alcohol**	0.042	1.042	0.795–1.366	0.764
**Phase angle (degree)**	0.266	1.304	0.996–1.708	0.054
**QOL (EQ-5D)**	−0.018	0.982	0.969–0.995	0.009
**CAT score**	0.046	1.048	1.013–1.084	0.007
**Gastrointestinal-related QOL**				
**Reflux**	−0.07	0.932	0.837–1.039	0.204
**Upper abdominal pain**	0.053	1.055	0.930–1.196	0.408
**Fullness**	0.113	1.119	1.028–1.220	0.010
**Constipation**	0.060	1.062	0.974–1.158	0.173
**Diarrhea**	−0.053	0.948	0.872–1.032	0.217
**Severity of constipation**				
**Constipation Scoring System score**	−0.007	0.993	0.925–1.067	0.855
**Neuropsychological examination**				
**MMSE score**	−0.089	0.915	0.854–0.980	0.011
**GDS-15 score**	0.096	1.101	1.026–1.182	0.008
**Health condition**				
**History of falls**	0.532	1.702	1.056–2.743	0.029
**History of day care use**	0.187	1.206	0.645–2.256	0.558
**Social frailty**	0.396	1.485	0.892–2.473	0.128
**Comorbidities**				
**Cerebral infarction/hemorrhage**	0.453	1.573	0.762–3.246	0.221
**Myocardial infarction**	0.849	2.337	0.887–6.157	0.086
**Hospitalization for heart failure**	−0.414	0.661	0.200–2.184	0.497
**Interstitial pneumonia**	0.166	1.180	0.502–2.777	0.704
**Hypertension**	0.145	1.156	0.726–1.843	0.541
**Diabetes mellitus**	0.489	1.631	0.966–2.755	0.067
**Osteoporosis**	0.064	1.066	0.661–1.720	0.793
**Sarcopenia**	1.549	4.708	3.031–7.311	<0.001
**Use of therapeutic agents**				
**Statin**	0.038	1.039	0.642–1.682	0.876
**Acid secretion suppressant**	−0.034	0.967	0.596–1.566	0.890
**Laxative**	0.037	1.038	0.601–1.793	0.894
**Steroid**	1.319	3.741	1.669–8.383	0.001
**Analgesic drugs**	0.721	2.056	1.158–3.650	0.014
**Antidementia drugs**	−0.387	0.679	0.165–2.795	0.592
**Number of oral medicines**	0.007	1.007	0.937–1.083	0.841
**Pulmonary function**				
**Restricted ventilatory impairment**	0.223	1.250	0.730–2.138	0.416
**Nutritional status**				
**Hypozincemia**	−0.008	0.992	0.551–1.786	0.979
**CONUT score**	0.037	1.037	0.867–1.241	0.690
**Dietary Variety Score**	0.011	1.011	0.912–1.119	0.840
**Oral function**				
**Oral frailty**	0.055	1.056	0.661–1.689	0.819
**EAT10 score**	0.068	1.071	1.017–1.127	0.009

CAT—chronic obstructive pulmonary disease assessment test; CI—confidence interval; CONUT—CONtrolling NUTritional status; EAT10—10-item Eating Assessment Tool; EQ-5D—EuroQol-five dimension; GDS-15—15-item Geriatric Depression Scale; MMSE—Mini Mental State Examination; OR—odds ratio; QOL—quality of life.

## Data Availability

Data supporting the findings of this study are available upon request from the corresponding author. The data are not publicly available due to privacy or ethical restrictions.
